# Langmuir-Blodgett monolayers holding a wound healing active compound and its effect in cell culture. A model for the study of surface mediated drug delivery systems

**DOI:** 10.1016/j.heliyon.2021.e06436

**Published:** 2021-03-14

**Authors:** Luciana Fernández, Ana Lucía Reviglio, Daniel A. Heredia, Gustavo M. Morales, Marisa Santo, Luis Otero, Fabrisio Alustiza, Ana Cecilia Liaudat, Pablo Bosch, Enrique L. Larghi, Andrea B.J. Bracca, Teodoro S. Kaufman

**Affiliations:** a Departamento de Física, Departamento de Química, Universidad Nacional de Río Cuarto, CONICET, Agencia Postal 3, X5804BYA, Río Cuarto, Argentina; b Grupo de Sanidad Animal, INTA Estación Experimental Agropecuaria Marcos Juárez, X2580, Marcos Juárez, Argentina; c Departamento de Biología Molecular, Universidad Nacional de Río Cuarto, Agencia Postal 3, X5804BYA, Río Cuarto, Argentina; d Instituto de Química Rosario (IQUIR, CONICET-UNR) and Facultad de Ciencias Bioquímicas y Farmacéuticas, Universidad Nacional de Rosario, Suipacha 531, S2002LRK, Rosario, Argentina

**Keywords:** Langmuir monolayers, Drug-carrier composites, Cell culture, Triclisine

## Abstract

Langmuir and Langmuir-Blodgett films holding a synthetic bioinspired wound healing active compound were used as drug-delivery platforms. Palmitic acid Langmuir monolayers were able to incorporate 2-methyltriclisine, a synthetic Triclisine derivative that showed wound healing activity. The layers proved to be stable and the nanocomposites were transferred to solid substrates. Normal human lung cells (Medical Research Council cell strain 5, MRC-5) were grown over the monomolecular Langmuir-Blodgett films that acted as a drug reservoir and delivery system. The proliferation and migration of the cells were clearly affected by the presence of 2-methyltriclisine in the amphiphilic layers. The methodology is proposed as a simple and reliable model for the study of the effects of bioactive compounds over cellular cultures.

## 1 Introduction

As the lipid fractions of biological membranes are essentially formed by different phospholipids, Langmuir [1, 2] and Langmuir Blodgett (LB) [3, 4, 5] films formed by amphiphilic compounds are extensively used as mimetic systems and as models to scrutinize cellular membrane processes. This technique has been applied in the study and development of different active compounds, such as antibacterial [6, 7] and antifungal [8] drugs, as well as anti-inflammatory [9], sedative [10], neuroactive drugs [11] and antitumor [12, 13] agents, among others.

Typically, these investigations are primarily based on the analysis of the interactions between biomolecules and the corresponding target active drugs that have been incorporated into the biomimetic membranes, forming vesicles or Langmuir [1] and Langmuir Blodgett [14, 15] films. In addition, Langmuir films have been used as models to study the incorporation of active compounds in cell membranes. For example, Fernandes et al. demonstrated the penetration of nano-penicillin G spheres within Langmuir monolayers formed by L-α-phosphatidylethanolamine, the principal phospholipid extracted from *E. coli* [16].

However, despite remarkable examples of cell culture over Langmuir, Langmuir-Blodgett and Langmuir-Schäfer layers [17, 18], to the best of our knowledge, few studies have been developed using monomolecular Langmuir-Blodgett films acting as biomimetic drug reservoir and delivery systems [11, 12, 19]. Higuchi et al. demonstrated that fibroblast L929 cells cultured on collagen adhered and spread better on synthetic polymeric films prepared by the LB method than on films obtained by the casting method [20]. Also, human interferon-β was produced by inoculation of fibroblast cells cultured on polymeric films obtained by the Langmuir-Blodgett technique [21]. Recently, Bhuvanesh et al. reported the building of artificial biomimetic structures, where the cell stem adhesion can be directed by the surface areal density of collagen type-4 in Langmuir films [17]. Similarly, hippocampal pyramidal neurons were cultured onto LB films of insulin with different surface packing density, showing that the neuron polarization through the activation of the Insulin-like Growth Factor-1 receptor can be selectively modulated by the lateral packing of insulin, organized as a monomolecular surface for cell growth [22]. Also, the study of the LB collagen deposition conditions on the adhesion and proliferation of Sprague-Dawley rat bone marrow mesenchymal stem cells revealed that highly oriented and collagen-abundant thin films facilitate cell adhesion and proliferation, turning the LB technique into a useful tool for tissue engineering [23].

**Figure 1:**
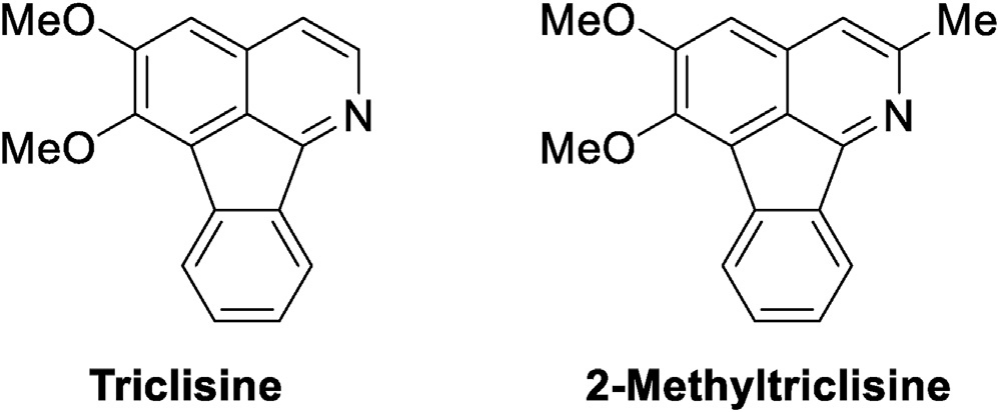
Chemical structures of the naturally-occurring azafluoranthene alkaloid triclisine and the synthetic 2-methyltriclisine (**MT**).

In this paper, we reported the incorporation of 2-methyltriclisine (**MT**), a synthetic Triclisine derivative (Figure 1), in stable Palmitic Acid (**PA**) Langmuir monolayers, and the transference of the composite monomolecular layers to solid substrates as Langmuir-Blodgett films. **PA** is utilized as an important component in therapeutic formulations [24], whereas natural Triclisine is a azafluoranthene alkaloid [25] which was isolated from the Amazonian vine *Triclisia gilletii* [26]. It has been reported that some naturally-occurring azafluoranthenes display relevant biological activities [27], as antifungal [28], anti-HIV [29], and antidepressant [30, 31] agents, and they have been patented as the active components of wound-healing preparations [32]. Furthermore, it is interesting that some plants of the genus *Triclisia*, or those producing azafluoranthenes, are ethnopharmacologically known for their healing properties [33]. In this sense Triclisine has been predicted as a potentially useful compound with various relevant pharmacological applications [24, 34].

We also reported the effect of the presence of **MT** in LB layers on the development of human normal lung fibroblast cells (Medical Research Council cell strain 5, MRC-5), cultivated over the monomolecular LB films, that acts as drug reservoir. The cell proliferation was noticeably affected by the presence of the synthetic Triclisine analogue in the amphiphilic layers.

## 2 Materials and methods

### 2.1 Chemicals and reagents

Ultrapure water was obtained from a Elga Classic equipment (resistivity 18 MΩ cm). Anhydrous solvents were prepared following conventional procedures [35] and stored in dry Young ampoules. Palmitic Acid and the remaining reagents were purchased from Sigma-Aldrich and used as received. 2-Methyltriclisine (**MT**, 5,6-dimethoxy-2-methylindeno[1,2,3-*ij*]isoquinoline) was synthesized as previously described [31] and stored at room temperature under vacuum. All solvents utilized were of HPLC quality; they were acquired from Sintorgan (Buenos Aires, Argentina). Palmitic Acid was purchased from Sigma-Aldrich and used as received.

### 2.2 Synthetic procedures and product characterization

The reactions were executed with oven-dried glassware, under dry nitrogen atmosphere. Flash column chromatography was carried out with silica gel 60 H, eluting with hexane/EtOAc mixtures, of increasing polarity under positive pressure. All new compounds gave single spots on TLC plates, developed in hexane/EtOAc and CH_2_Cl_2_/toluene solvent systems. The spots were detected by UV-light irradiation (254 nm), followed by spraying with *p*-anisaldehyde/sulfuric acid reagent in EtOH or ethanolic ninhydrin and careful heating for improving selectivity.

The compounds were characterized by FTIR spectroscopy employing a Shimadzu Prestige 21 spectrophotometer, and by NMR spectroscopy using a Bruker Avance 300 MHz spectrometer. In case of solids, their melting point was also determined in a Leitz model 350 hot-stage microscope. The microwave-assisted reactions were carried out in a CEM discovery synthesis microwave oven.

### 2.3 Langmuir and Langmuir-Blodgett films

A Nima Technology Model 611 Langmuir-Blodgett trough was used for monomolecular monolayers formation and transference to freshly cleaved hydrophilic mica sheets, used as solid substrates. The surface pressure was measured using the Wilhelmy plate method, and the subphase temperature was kept constant (298 K) by the circulation of thermostated water in the trough. Prior to spreading over the subphase the chloroform solutions of Palmitic acid (50 μL, 3 × 10^−3^ M), blank control isotherms were run in order to confirm the cleaning of the water surface. In all cases, 10 min were allowed to pass to allow chloroform evaporation. Mixed Palmitic acid-2-methyltriclisine (**PA-MT)** monomolecular layers were formed from mixed chloroform solutions (**PA**/**MT** molar ratio 7/1). This ratio allows to obtain reproducible and stable monolayers in the air-water interface. A larger **MT** concentration in the bidimensional liquid composite can drive to the MT aggregates formation, affecting the stability of the Langmuir film. On the other hand, low **MT** concentration precludes the detection of observable changes in the surface pressure-area isotherms that evidence the presence of the active compound as solute in the mixed monolayer. The compression and expansion barrier speed was set at 50 cm^2^/min for all monolayers. LB monolayers were obtained by the vertical transference method at 5 mm/min holding a constant surface pressure of 27 mN m^−1^. At this pressure the monolayers are in liquid-condensed phase, and it is lower than the monolayer collapse pressure, allowing the successfully transfer of pure **PA** and **PA-MT** monolayers to mica solid substrate. Only films having transfer ratios of 1.0 ± 0.1 were used for the further biological assays.

### 2.4 Atomic force microscopy (AFM)

The AFM characterization of the Langmuir-Blodgett films was performed using an Agilent 5500 SPM microscope (Agilent Technologies, Chandler, AZ, U.S.A.) working in Acoustic AC Mode in a stationary dry-air atmosphere. Commercial silicon cantilever probes, with an aluminum backside coating and nominal tip radius of 10 nm (MikroMasch, NSC15/Al BS/15, spring constant in the range 20–75 N/m), were employed just under their fundamental resonance frequencies of about 325 kHz. The images were treated and analyzed using Gwyddion, an open source software for the visualization and analysis of scanning probe microscopy data.

In order to avoid any post processing artifacts, the rough surface analysis was performed using the images as obtained; only a standard background correction was applied to remove the piezo movement and sample tilt. The reported values were obtained using the statistical quantities tool in Gwyddion. The reported surface roughness parameters are the average of different images with the same area (0.5 × 0.5 μm) and resolution (512 × 512 pixels, and line rates of 1 Hz).

### 2.5 Computational methods

In order to access the spatial arrangements and the relative formation energies of both, **PA** clusters and **PA-MT** bidimensional liquid composite in Langmuir films, a high-level ab initio modeling was carried out, using the electronic Density Functional Theory (DFT). The DFT calculations were performed with the Quantum Espresso package [36, 37], which operates on periodically repeated unit cells and plane waves bases. The calculations were made with Vanderbilt ultrasoft pseudopotentials and Perdew Burke Ernzerhof (PBE) simplified generalized gradient approximation [38] exchange-correlation functional, using a plane wave cut-off of 750 eV at the Γ–point, with a convergence threshold for the total energy set to 0.0001 eV. Surfactant-surfactant interactions greatly influence the physicochemical properties of Langmuir monolayers; therefore, the dihydrogen (intermolecular van der Walls) interactions critically contribute to the packing energy, and to the monolayer formation [39]. As consequence, the DFT-D2 [40,41] approach was implemented in the calculations to include this type of forces.

The crystallographic structures of **PA** Langmuir monolayers were created based on the optimized structures of Toledano et al. [39] and conserving the area per molecule found experimentally here. Each unit cell contains two **PA** molecules with approximately 1.4 *a/b* ratio, which has been related to the herringbone (HB) structure [42]. The **PA-MT** Langmuir monolayers were generated from a super-cell contained 8 molecules of **PA** where one of them was replaced by the **MT** molecule; this procedure enables to reproduce the experimental **PA**/**MT** molar ratio used in composite Langmuir films. The atomic positions of **MT** inside **PA-MT** structure were optimized using Broyden-Fletcher-Goldfarb-Shanno (BFGS) algorithm [43] with a 0.001 (a.u) threshold of convergence. All structures have 7Å of vacuum in z direction to avoid self-interactions between monolayers. The surfactant-subphase interaction is considered constant in both, **PA** and **PA-MT** Langmuir films.

### 2.6 Normal human lung cells (MRC-5) culture

The MRC-5 cell line (derived from human lung tissue, obtained from cells bank, Departamento de Biología Molecular, Universidad Nacional de Río Cuarto) was used to investigate the proliferation and migration of the cells when they are cultured on the different monomolecular films. Before cell culture, LB films over mica substrate were carefully immersed three times (3 min each), in an antibiotic-antimycotic solution (Gibco®, Invitrogen, Grand Island, New York, USA). Subsequently, the films were washed three times by immersion in sterile phosphate buffer solution (PBS) and irradiated with short wavelength UV-light (254 nm) for 60 min. AFM images of the LB films before and after cleaning and sterilization processes didn't reveal noticeable differences in the topographic images, indicating that the LB films remained without alterations. The Langmuir-Blodgett monolayers over mica substrates were separately located on the bottom of a 60 mm culture dish. Then, MRC-5 cells were seeded on each surface at a density of 5 × 10^6^ cells per plate.

The cell culture was carried out in complete Dulbecco's Modified Eagle Medium (DMEM, Gibco® Invitrogen, Grand Island, New York, USA) supplemented with 10% of fetal bovine serum (FBS, NATOCOR®, Córdoba, Argentina) and the antibiotic-antimycotic solution at 37 °C in a humidified atmosphere of 5% CO_2_ in air. The morphology of the cells attached on each surface was observed after different times of culture (24, 48 and 72 h) in an inverted microscope (Nikon Ti–S 100, Nikon Corp., Tokyo, Japan) and pictures were taken using a Nikon digital camera (Nikon Corp., Tokyo, Japan). The experiments were done in triplicate.

### 2.7 Hoechst staining

Hoechst 33258 is a fluorescent nucleic acid stain commonly used in the study of nuclear cell morphology and structure, and to identify cells in mitosis. For the Hoechst staining study, the MRC-5 cells were cultured on LB films treated as described in the previous section. The slides were rinsed with PBS, fixed overnight in 100% methanol, permeabilized for 10 min with 0.1% Triton X-100, and immersed in a Hoechst 33258 (Sigma-Aldrich Ltd., St. Louis, MO, USA) solution (1 μg/mL in PBS). The pictures of the stained cells were taken with a Nikon digital camera mounted on a Nikon Ti–S 100 inverted microscope equipped with epifluorescence illumination (excitation at λ = 360 ± 40 nm and fluorescence emission at λ = 460 ± 50 nm). As it is known, the percentage of mitotic cells was estimated by counting the number of mitotic nuclei over the total nuclei.

### 2.8 Wound healing assay

For the wound healing assay, MRC-5 cells were grown as described in section 2.6, in the bottom of 60 mm culture dishes. After reaching 100% confluence, a wound was created in the monolayer cells with a sterile tip. Subsequently, the **PA** and **PA-MT** monolayers deposited over mica substrate were contacted with the previously made wounded monolayers. Photographic images were taken after 5 h of exposure and the width of the wounds, which indicates cell migration, was quantified using the Image J v.1.45s software (National Institutes of Health, Bethesda, USA) [44].

## 3 Results and discussion

### 3.1 Synthesis of **MT**

**Scheme 1:**
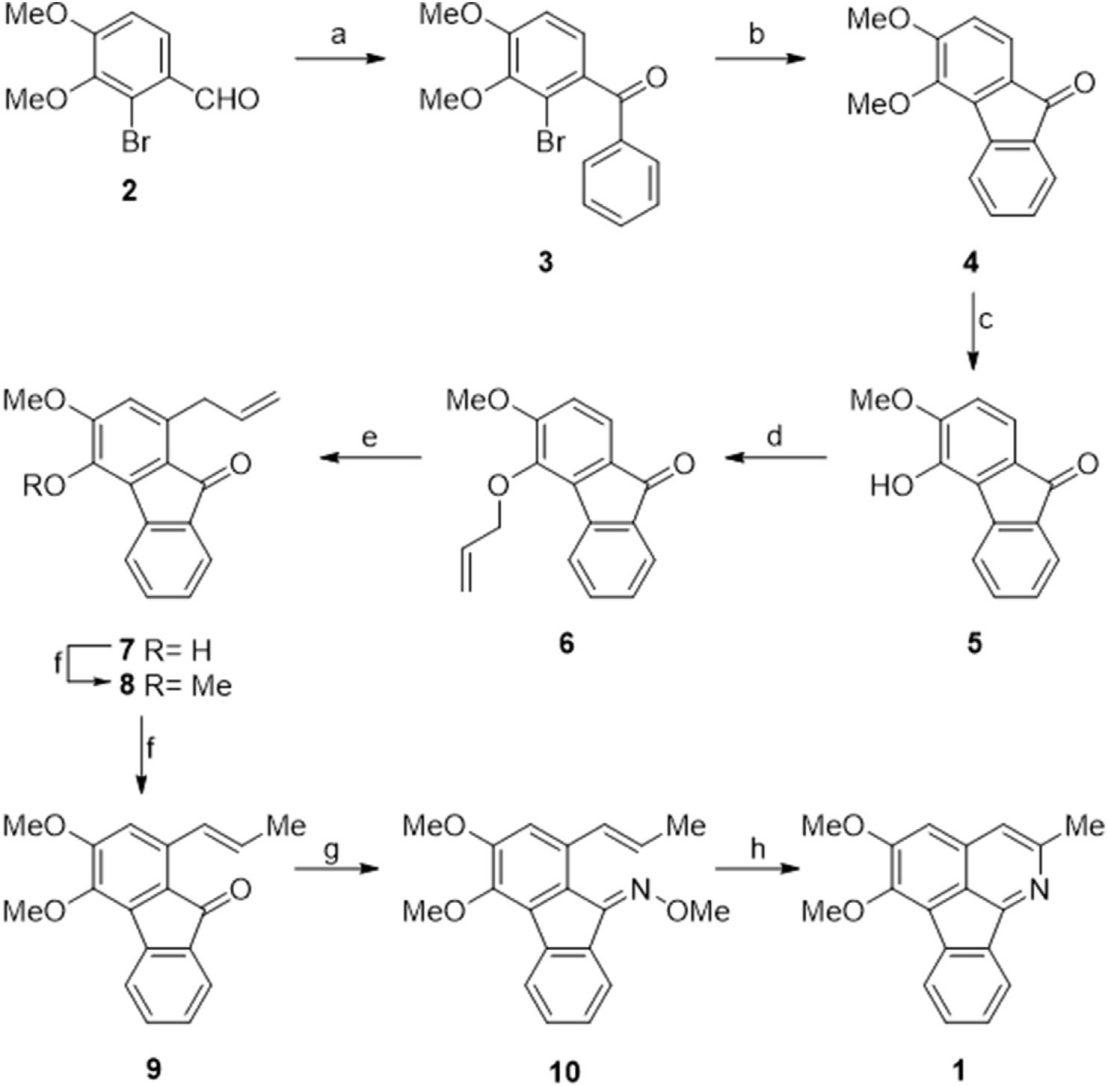
Synthesis of 2-methyltriclisine (**MT**, **1**). *Reagents and conditions.* a) 1. PhMgBr, THF, 0ºC→RT (94%); 2. PDC, CH_2_Cl_2_, RT, 15 h (92%); b) Pd(PPh_3_)_4_, Davephos, KAcO, K_2_CO_3_, DMA, 110 °C, 22 h (88%); c) NaH, EtSH, DMF, 50ºC, 17 h; d) H_2_C = CHCH_2_Br, K_2_CO_3_, EtOH, reflux, 90 min (52%, over two steps); e) 1. 1,2-Cl_2_-C_6_H_4_, reflux, 12 h (80%); 2. MeI, K_2_CO_3_, EtOH, reflux, 2 h (92%); f) PdCl_2_(MeCN)_2_, CH_2_Cl_2_, reflux, 60 h (90%); g) H_2_NOMe.HCl, NaOAc, EtOH, 2 h (95%); h) 1,2-Cl_2_-C_6_H_4_, MW (115 W, 180ºC), 60 min (81%).

The synthesis of 2-methyltriclisine (**MT**, **1**) was carried out in ten steps and 21% overall yield from the bromoaldehyde **2**, easily available from isovanillin by successive *ortho*-bromination and Williamson *O*-methylation reactions (Scheme 1) [31]. In brief, for the synthesis, compound **2** was treated with PhMgBr in THF, and the resulting benzhydrol (94% yield) was oxidized with pyridinium dichromate (PDC) to the benzophenone **3** in 92% yield. The latter was then submitted to cyclization under Pd(PPh_3_)_4_ catalysis in dimethylacetamide (DMA), employing Davephos as a ligand and a mixture of KAcO and K_2_CO_3_ as bases.

The resulting fluorenone derivative **4** (obtained in 88% yield) was then selectively demethylated with sodium ethylmercaptide prepared *in situ*, to afford **5** and the free phenol was converted into the allyl derivative **6** in 52% overall yield, with allyl bromide under Williamson conditions. Next, the allyl ether **6** was subjected to a Claisen rearrangement in 1,2-dichlorobenzene, affording 80% yield of compound **7**, which was *O*-methylated with MeI and K_2_CO_3_ in refluxing ethanol, to provide the key intermediate **8** in 92% yield.

In turn, a Pd(II)-mediated allyl to propenyl isomerization with PdCl_2_(MeCN)_2_ in refluxing CH_2_Cl_2_ was put in place, to obtain the styryl-fluorenone derivative **9** in 90% yield. To complete the synthesis, the ketone was transformed into the related *N*-methoxy oxime in 89% yield, and the latter was finally subjected to a microwaves-assisted 6π-electrocyclization, furnishing 2-methyltriclisine (**1**) in 81% yield.

### 3.2 **PA** and **PA-MT** composite Langmuir films

**MT** doesn't form a monomolecular monolayer when it is spread on a water surface, and remains as aggregated islands in the water-air interface. This is an expected result for azafluoranthene alkaloids including **MT**, despite the presence of methoxy groups, that confer to the structure an amphiphilic character. Further, the presence of the planar π-conjugate fused rings in the molecular structure of **MT** prompts for the formation of aggregates [27]. On the contrary, when **MT** was co-spread with **PA** from a mix solution in chloroform, no islands nor aggregate formation were observed in the water-air interface after solvent evaporation.

**Figure 2:**
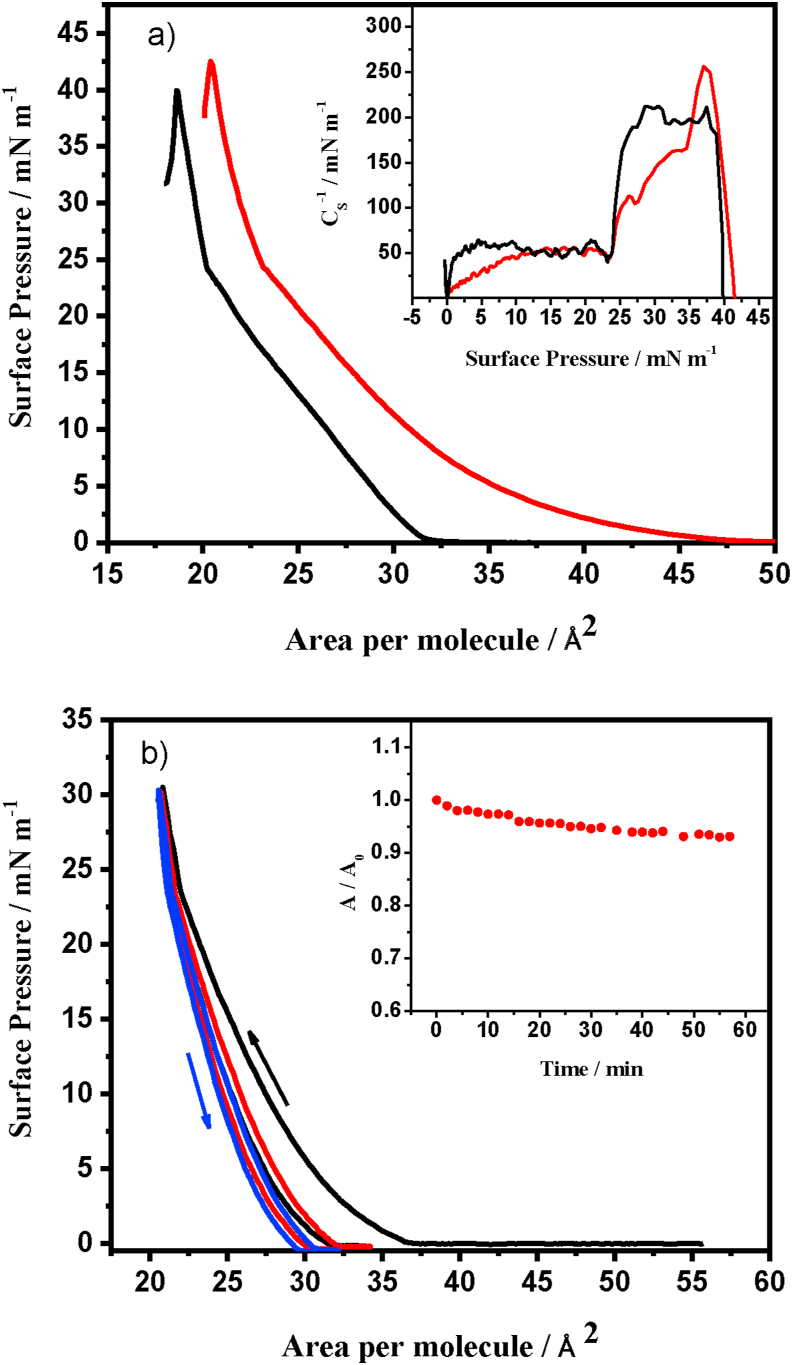
a) Surface pressure-area isotherms of the pure **PA** (black) and the **PA-MT** monolayers (red) at the air-water interface at 25 °C. The inset shows the Compression Modulus (C_s_^−1^) versus Surface pressure graph for the pure **PA** (black) and **PA-MT** (red) monolayers. b) Compression-expansion cycles of the **PA-MT** Langmuir monolayers on the water subphase at high surface pressures and 25 °C. The first (black), second (red) and third cycle (blue) are shown. The inset shows the A/A_0_ versus time graphs for the **PA-MT** monolayers recorded at 27 mN m^−1^.

The surface pressure-Area (π-A) isotherm of the mixed **PA-MT** monolayer is shown in Figure 2a, along with the π-A isotherm obtained under the same conditions for a **PA** monolayer, for comparison purposes. Both isotherms exhibit the three characteristic regions corresponding to gaseous (G), liquid-expanded (LE) and liquid-condensed (LC) phases [19]. For the **PA** isotherm, the pressure transitions among the different regions are consistent with those reported in the literature [45, 46]. The shape of the isotherms showed in Figure 2a allows us to infer that the molecules present at the interface gradually accommodate without suffering conformational changes during compression, and displaying a direct transition from the gas phase to the condensed phase [47]. This fact is interpreted as an increase in the surface concentration and a decrease in the tilt angle of the hydrocarbon chains of the **PA** amphiphilic compound, as the available area per molecule decreases. The inset in Figure 2a shows that for **PA** the π-A curve displays abrupt changes in the compression modulus (defined as C_s_^−1^ = -A(δπ/δA)), which correspond to the phase transitions between G, LE and LC regions. On the other hand, the phase transitions in surface pressure-Area isotherm of the mixed **PA-MT** monolayer are smoother, as can be seen in the C_s_^−1^ – π relationship exhibited in the inset of Figure 2a. For example, in the range 0–10 mN m^−1^ the value of the compression modulus increases gradually for the mixed system, while for **PA** pure monolayer the changes in compression modulus are abrupt. The same is observed between 25 and 35 mN m^−1^ surface pressures. This fact is in agreement with presence of **MT** as solute in the bidimensional liquid formed by **PA** monolayer. It can be proposed that the presence of **MT** in the **PA** monolayer partially interferes with the interactions between hydrocarbon chains and hinders the tilt angle changes, generating more gradual variations in the pressure-Area isotherm.

On the other hand, a displacement of the **PA-MT** mixed film isotherm to larger areas is observed, with regards to the isotherm of pure **PA**. As in Figure 2a the surface pressure is expressed as a function of the occupied area by the **PA** molecules, the shift observed in the **PA-MT** isotherm clearly exposed the presence of **MT** molecules at the interface. There are only slight changes in the shape of the curve; only an almost continuous transition between the G and LE regions is distinguished (see inset in Figure 2a) and a small increase in the value of the collapse pressure from 40 to 42.5 mN m^−1^ is also detected, as consequence of the presence of **MT** in the monomolecular monolayer.

The A_0_ parameter, which denotes the area occupied by a molecule in a situation of maximum packing [46], can be obtained from the π-A isotherms by extrapolating the slope of the condensed phase to zero-pressure. This parameter provides quantitative information on the dimensions and shape of the monomolecular arrangement at the interface. For the **PA** film, an occupied area of 22.5 Å^2^ per molecule was obtained. This area is similar to the values reported in literature [45, 46], and it is consistent with the calculated geometric projection for **PA** molecule (A_0_ = 22.48 Å^2^). On the other hand, for the isotherm of **PA-MT** mixed monolayer, as the surface pressure is expressed as a function of the occupied area by **PA** molecules, an A_0_ apparent value of 25.5 Å^2^ was obtained, which is larger than the obtained for pure **PA** films. Using this value and the concentration relationship in the spreading solutions, an area occupied by **MT** molecule of 20.9 Å^2^ was calculated, supposing that all **MT** molecules are located in the interface [12]. This value is in close agreement with the projected area (22.5 Å^2^) of the minimum box that can contain one **MT** molecule, calculated by quantum mechanics methods.

Furthermore, we evaluated the stability of the **PA** Langmuir monolayer holding **MT**, when it is subjected to repetitive compression-expansion cycles. As displayed in Figure 2b, the first compression step (black line) reached a surface pressure of 30 mN m^−1^, where a liquid-condensed phase is formed. When the barrier is open in the subsequent expansion step, a small hysteresis is observed due to the interaction between the amphiphilic molecules in the monolayer [48]. However, in the subsequent compression-expansion cycles the isotherms are almost superimposed, evidencing that there is no loss of material and/or multilayer formation. Moreover, the projected area per molecule obtained by extrapolation of the slopes observed in the condensed phase to zero-pressure, is nearly constant for all different cycles. Equal results were observed for pure **PA** monolayers.

Since the stability of Langmuir films is a fundamental requirement for their transfer to solid substrates with satisfactory transfer ratios, the area occupied per molecule (A) was recorded during one hour at a constant surface pressure of 27 mN m^−1^. The inset of Figure 2b shows the area occupied per molecule relative to the initial area (A/A_0__i_). A small variation of less than 5% was observed in the area occupied by the monolayer, which indicated again that there is no loss of material by dissolution or film collapse. These results are very similar to those obtained for pure **PA** films, highlighting that the presence of **MT** doesn't affect the stability of the monolayers, and that the active compound remains as a solute in the two-dimensional liquid.

**Figure 3:**
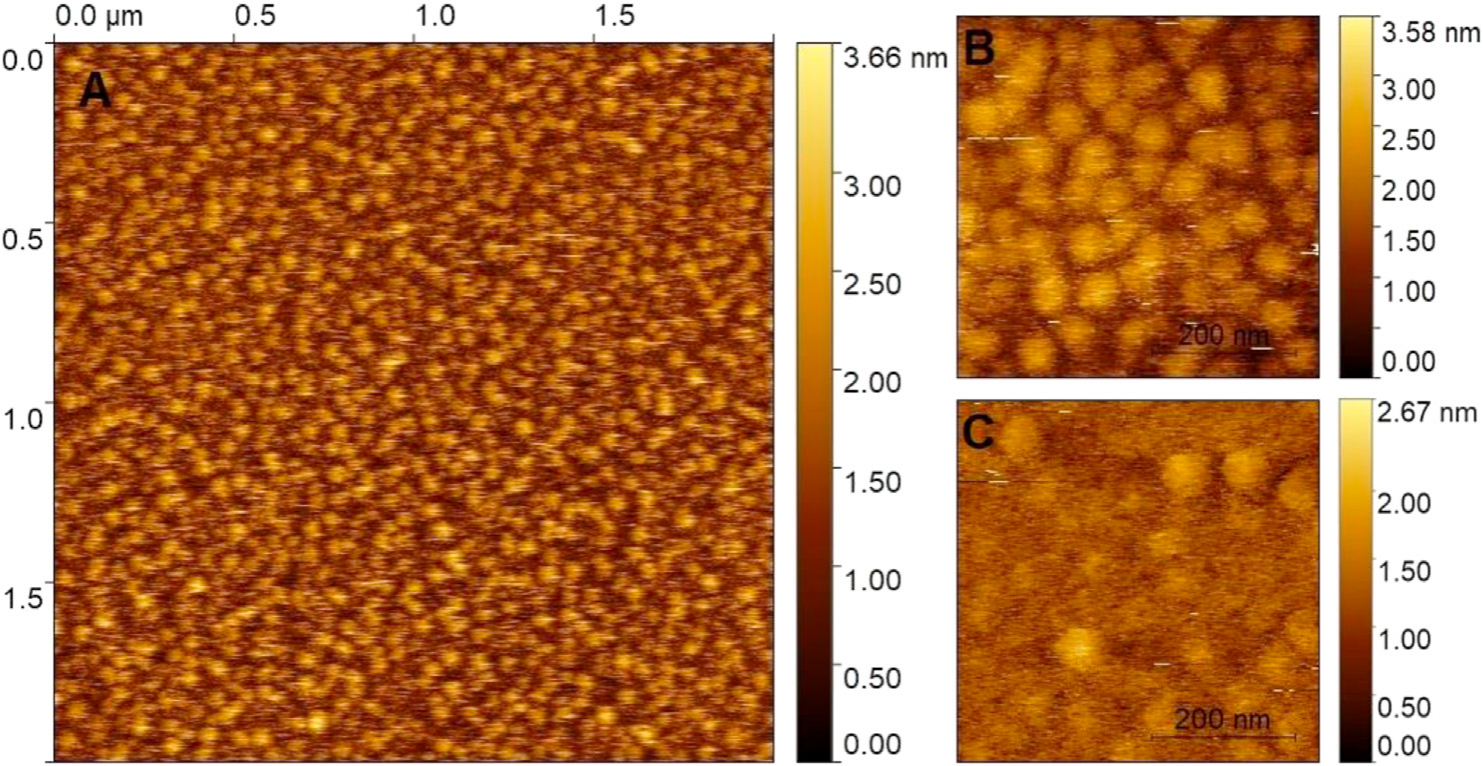
Topographic AFM images of Langmuir-Blodgett films transferred on mica at 27 mN m^−1^ and 25 °C. (A) **PA-MT**; (B) High resolution (0.5 × 0.5 μm) of **PA-MT** and (C) pure **PA** films.

Both, the pure **PA** and **PA-MT** monolayers were successfully transferred to a mica solid substrate at a surface pressure of 27 mN m^−1^ (liquid condensed phase). Adequate transfer ratios of 1.0 ± 0.1 were obtained, and the surface of the so-obtained Langmuir-Blodgett films was analyzed by AFM. Figure 3 shows the surface topography AFM images of the **PA** and **PA-MT** monolayers on mica.

In both cases, it is observed a nearly full coverage of the surface with no (or undetectable) pinhole defects, which is an indication of an efficient transfer of the film formed at the air/water interface to the mica substrate. In addition, the surface roughness (Sa), and the RMS (root-mean-square) surface roughness (Sq) measured for samples prepared from pure **PA** (Sa: 488 ± 172 pm; Sq: 607 ± 194 pm) and the mixture **PA-MT** (Sa: 503 ± 183 pm; Sq: 642 ± 201 pm) remain almost constant on the monolayers deposited over mica. These results suggested that the presence of the drug in the monolayer doesn't alter the packing order of **PA** in the film.

### 3.3 Computational analysis of **PA** and **PA-MT** Langmuir monolayers

**Figure 4:**
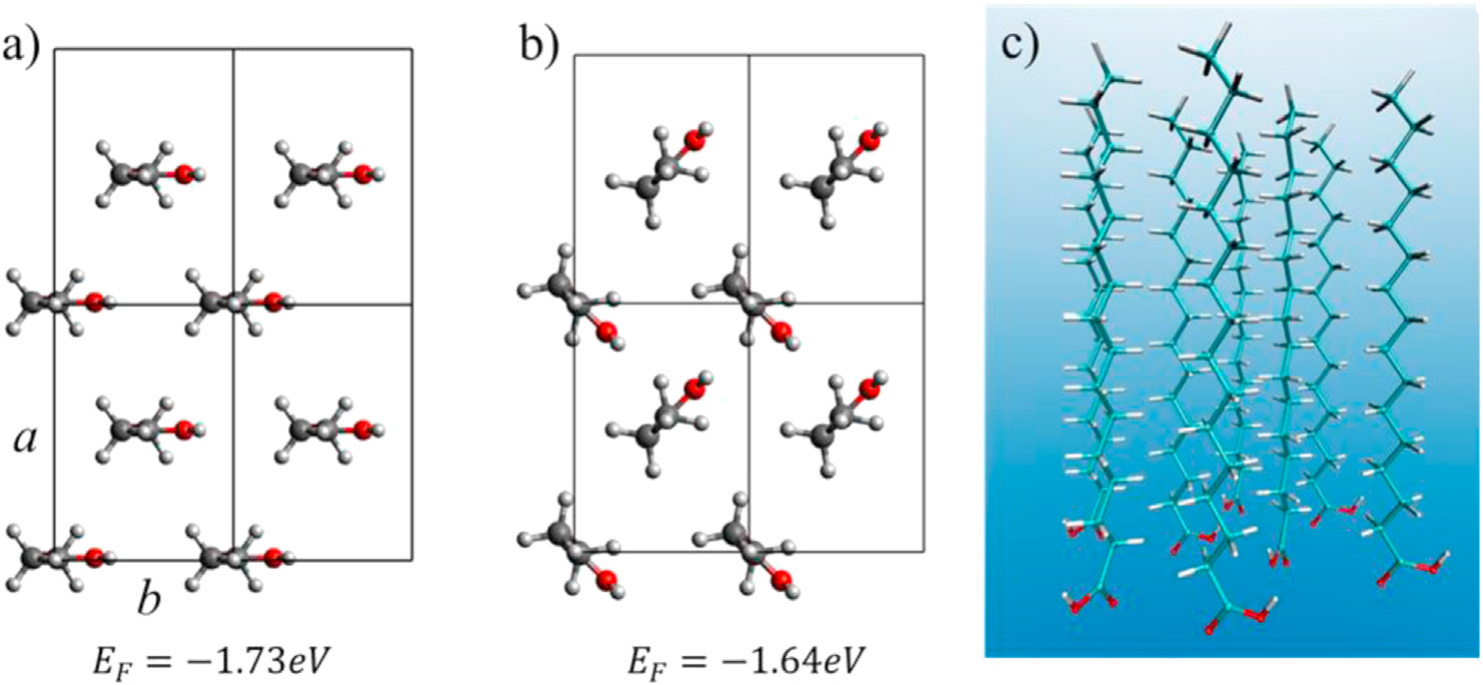
Top view of the unit cell for each structure a) PAp parallel **PA** monolayer and b) PAa antiparallel **PA** monolayer. c) Longitudinal view of PAa.

The computational simulations of pure surfactant clusters and bidimensional liquid composites in Langmuir films using ab initio quantum chemistry methods is a challenging task, due to the complexity of the interactions present in the Langmuir monolayers, and to the large number of atoms that contains their long amphiphilic chains [39]. Here we reported the results of the theoretical studies using DFT to describe the structures underlying both, **PA** and **PA-MT** monolayers. We first carried out the simulation of **PA** monolayers, which will be the basis for the drug incorporation. We built two different structures for the **PA** monolayer with the same unit cell, whose parameters are *a = 8.020Å* and *b = 5.606Å,* and two molecules per cell. This preserved the experimentally found area per molecule of *22.5 Å*^*2*^ and the ratio *a/b = 1.43* associated with HB structures. We will call PAp to the structure where the **PA** molecules are aligned parallel to each other (see Figure 4a); and PAa, to the structure where the **PA** molecules align antiparallel (see Figure 4b).

The formation energy per **PA** molecule obtained by DFT calculations is reported under each structure in Figure 4. The negative values imply that both structures are possible in the formation of stable monolayers. Moreover, since the difference in formation energy is only 0.09 eV, which is in the order of the precision of the calculations [39], both structures coexist, as it is expected in the fluidic Langmuir monolayer, which has characteristics of bidimensional liquid [19].

**Figure 5:**
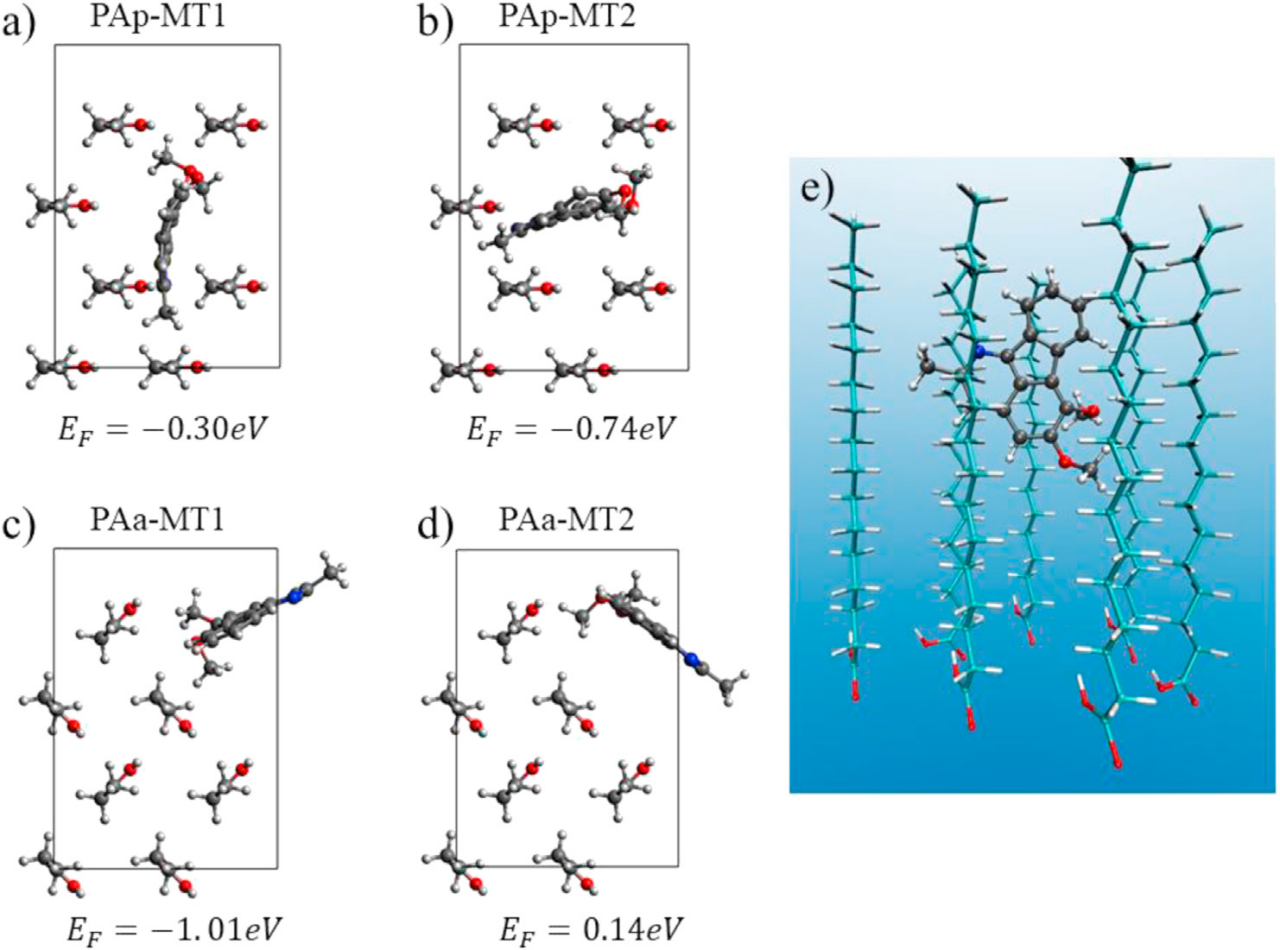
Top view of the unit cell for each structure of **PA-MT** monolayer: a y b) PAp-MT monolayer with two different orientations of **MT** and c y d) PAa-MT monolayer with two different orientations of **MT**. e) Longitudinal view of PPa-MT1.

Once the formation energies of the pure **PA** monolayers were obtained, new structures were assembled for the study of the **PA-MT** monolayer. Starting from PAp and PAa structures, the unit cell was replicated to form a super-cell where 8 **PA** molecules can be included. In the new super-cell, we placed seven **PA** molecules and one **MT** molecule, thus the experimental 7/1 ratio (**PA/MT**) in the Langmuir composite film is reproduced. The inclusion of the **MT** molecule was carried out proposing two possible orientations in each initial **PA** structure (Parallel or Antiparallel, see Figure 5). The composite structures were labeled as PAp-MT1, PAp-MT2, PAa-MT1 and PAa-MT2, depending on whether parallel **PA** or antiparallel **PA** is used as the initial structure, and the number denotes different **MT** orientation. The formation energy is calculated by the expression $E_{Formation}=E_{PA-MT}-7xE_{PA}-E_{MT}$, where $E_{PA-MT}$ is the total energy of **PA-MT** monolayer, $E_{PA}$ y $E_{MT}\;$ the energy of **PA** and **MT** molecules in vacuum, respectively. This subtraction cancels the contribution of surfactant-subphase interactions which are considered constant in both, **PA** and **PA-MT** Langmuir films. The formation energy per molecule in the super-cell is reported under each structure in Figure 5.

From the comparison of the formation energies of each structure, it is observed that the antiparallel PAa-MT2 structure holds positive formation energy, making its occurrence unlikely in the film structure. On the other hand, in both, parallel (PAp-MT1, PAp-MT2) and antiparallel (PAa-MT1) arrangements, the composite has favorable formation energies with the PAa-MT1 structure being the one with the lowest energy. This is in agreement with the formation of a stable bidimensional solution in the composite Langmuir monolayer, as it was experimentally observed. Also, the calculated negative energy for the composite formation is in concordance with the absence of islands or dominions originated by molecular segregation in the monolayer film. Both, LB expansion-compression experiment and AFM images, showed the formation of stable and homogeneous bidimensional composite, where the **MT** solute can be accessible in a surface mediated drug delivery system.

### 3.4 Normal human lung cells (MRC-5) culture

**Figure 6:**
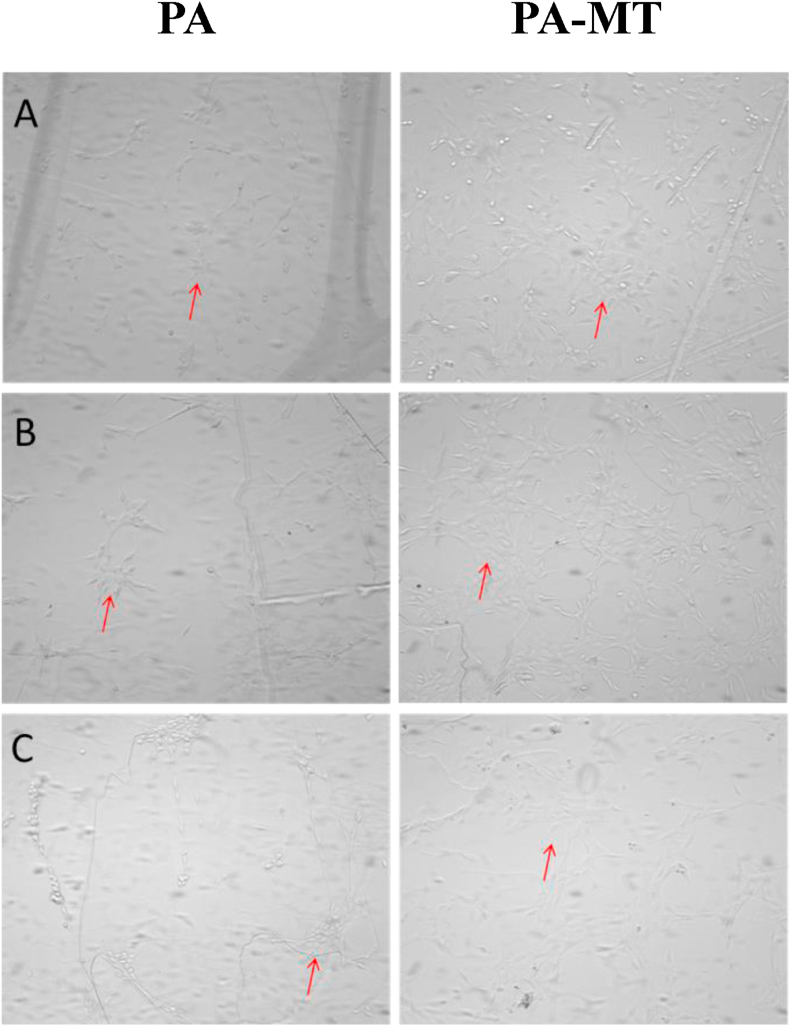
MRC-5 cells on either **PA** (left) or **PA-MT** (right) Langmuir-Blodgett surfaces, after a culture of (A) 24 h; (B) 48 h and (C) 72 h. Phase contrast images (magnification × 200). The arrows indicate culture cells with typical fibroblast morphology. Bacterial and/or fungal contamination is not evident.

With the purpose of to analyze the effects over a cell culture of the presence of **MT** as solute in the LB monolayer, the human fibroblast cell line MRC-5 was grown over both, pure **PA** and **PA-MT** monomolecular LB films formed on a mica substrate. The cell morphology was evaluated by phase-contrast microscopy after 24, 48 and 72 h of exposition to the surfaces. As shown in Figure 6, the MRC-5 cells were able to adhere to both, the **PA** and to **PA-MT** surfaces, while retaining the tapered cell morphology typical of cells of fibroblastic origin (Figure 6, red arrows). This effect was more evident after 48 and 72 h of exposure.

**Figure 7:**
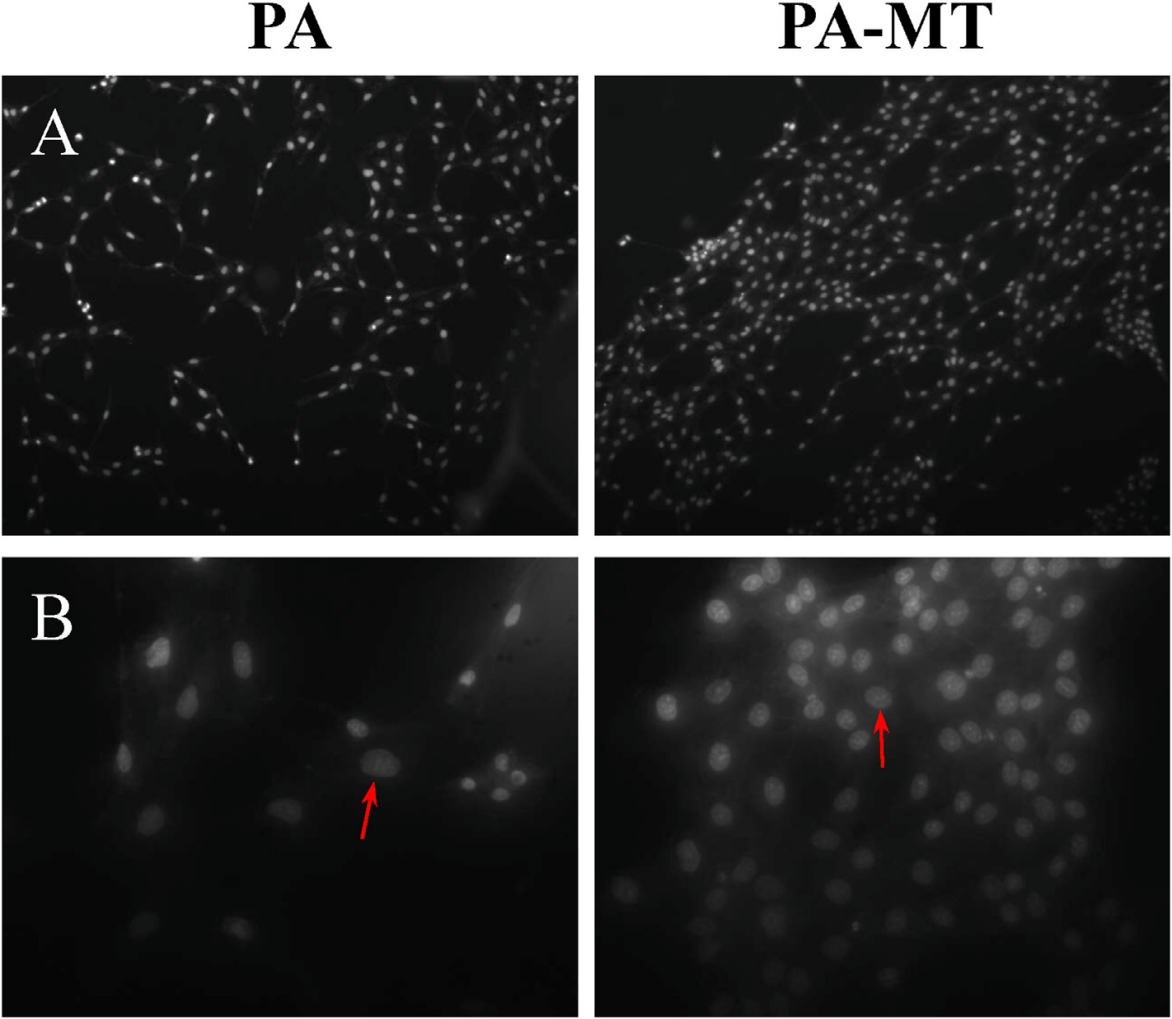
Fluorescence images of MRC-5 cells stained with Hoechst, after 72 h culture on Langmuir-Blodgett surfaces of either pure **PA** (left) or **PA-MT** (right). (A) Magnification: 200×; (B) Magnification: 800×. The arrows indicate normal nuclei morphology. Bacterial and/or fungal contamination is not evident.

Nuclear morphology is one of the most essential parameters to evaluate and analyze cytotoxicity mechanisms. Therefore, in order to assess whether pure **PA** and **PA-MT** monolayers was able to induce alterations in the genetic material of the MRC-5 cells, the morphology of their nuclei, exposed to the different monolayers during 72 h, was evaluated by the Hoechst staining method. As shown in Figure 7, the nuclei of the cells adhered to the pure **PA** and **PA-MT** surfaces remained without morphological alterations (control cells were grown under the same conditions, on the bottom of a culture dish).

It was remarkably interesting to notice that after 72 h of culture the count of nuclei was around twice as higher when the MRC-5 cells were grown over **PA-MT** monolayers, compared to those grown over the pure **PA** monolayer. As proven by the AFM images of the different LB monolayers, there are no noticeable physical differences between the LB **PA** films, when they are loaded or not with **MT**. Thus, the larger MRC-5 cell proliferation observed in the **PA-MT** monolayer could reflect a proliferative effect of the presence of **MT** as a solute in the amphiphilic layer.

As was already mentioned, plants producing azafluoranthenes and preparations containing the latter have been employed or registered for wound-healing purposes [32, 33]. Thus, it seemed interesting to study the effect of the presence of **MT** as a solute in the **PA** monolayer on cell migration when MRC-5 fibroblasts are in contact with Langmuir-Blodgett films. The wound-healing assay is a simple method that mimics cell migration during wound healing *in vivo*. It has been reported that the assay is suitable not only for studies involving cell–cell interactions on cell migration, but also for the analysis of the effects of its regulation by cell interaction with the extracellular matrix and soluble factors [49, 50]. The assay procedure involves the formation of a “wound” in the cell monolayer and the capture of images at regular intervals, from the onset and during the cell migration process that closes the wound, to quantify their migration rate. Although the wound-assay is not an exact duplication of cell migration *in vivo*, in the case of fibroblasts the cells migrate into the wound as loosely connected populations, mimicking the behavior of these cells during the *in vivo* migration process [51].

**Figure 8:**
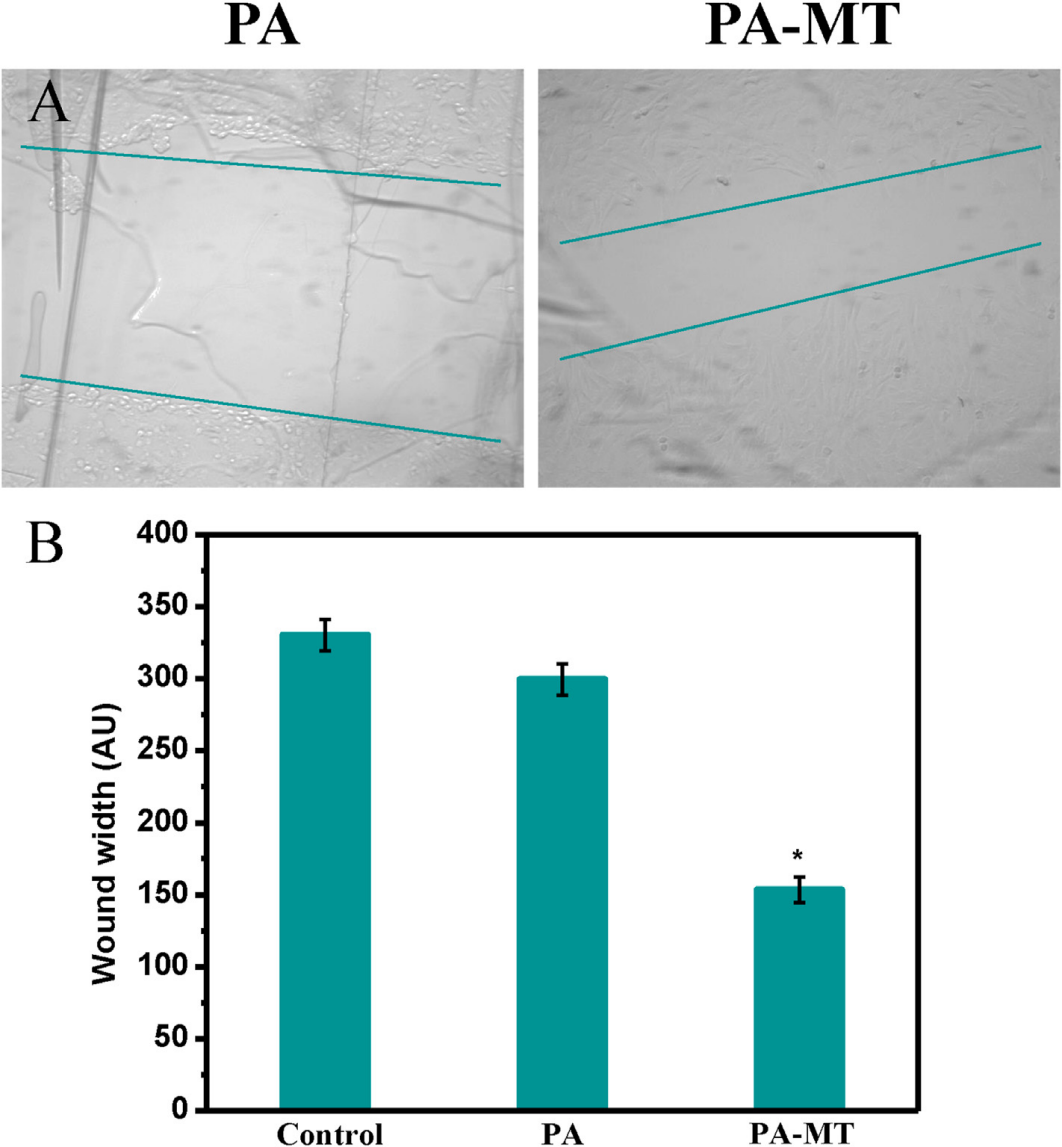
Migration of MRC-5 cells exposed to different surfaces during 5 h, analyzed by the wound assay at an image amplification of 200×. (A) Images of the width of the wounds of cells under different surfaces, including pure **PA** (left) and **PA-MT** (right). (B) Quantification of the width of the wounds [performed with the ImageJ program and expressed in arbitrary units (AU)]. The values correspond to three independent experiments. ∗*p* < 0.05 *vs*. **PA**.

The images of MRC-5 cultures when they were in contact with both, the **PA** and **PA-MT** surfaces were compared. As shown in Figure 8A, the MRC-5 cells that were exposed to **PA** surfaces for 5 h exhibited a wider wound with regard to the cells in contact with **PA-MT**. The corresponding quantifications (performed with the Image J software) are detailed in Figure 8B, along with the control in absence of LB films. Thus, it can be suggested that the presence of **MT** in the medium, as a solute in the amphiphilic layer, has a positive effect not only on cell proliferation as shown by Hoechst staining, but also on the migration of MRC-5 human fibroblasts.

## 4 Conclusions

This study showed that the growth of cells seeded on an amphiphilic monolayer holding an active compound as a solute in the two-dimensional liquid, generated by the Langmuir-Blodgett technique, can be proposed as a simple and reliable model for the study of the effects of bioactive compounds over cell cultures. It has been demonstrated that Palmitic acid Langmuir monolayers are able to incorporate the synthetic 2-methyltriclisine and that the resulting stable layers can be transferred to a mica substrate, creating a nanocomposite film that, acting as a drug reservoir and delivery system, contains the active compound as solute.

The Hoechst staining and the wound-healing assay demonstrated that proliferation and migration of MRC-5 cells (human fibroblasts) are beneficially affected by the presence of 2-methyltriclisine in the amphiphilic layers, converting this molecule into a potential candidate for wound-healing formulations, in the role of their pharmacological active ingredient.

## Declarations

### Author contribution statement

Luciana Fernández; Ana Lucía Reviglio; Gustavo M. Morales; Fabrisio Alustiza; Ana Cecilia Liaudat; Pablo Bosch; Enrique L. Larghi; Andrea B. J. Bracca; Daniel A. Heredia; Teodoro S. Kaufman: Performed the experiments; Analyzed and interpreted the data.

Marisa Santo; Luis Otero:Conceived and designed the experiments; Wrote the paper.

### Funding statement

Dr. Luis Otero was supported by 10.13039/501100003074Agencia Nacional de Promoción Científica y Tecnológica (PICT 2017-0524) and 10.13039/501100007868Secretaría de Ciencia y Técnica, Universidad Nacional de Río Cuarto (SECYT-UNRC18/C297) Teodoro S. Kaufman was supported by 10.13039/501100003074Agencia Nacional de Promoción Científica y Tecnológica (PICT 2017-0149).

### Data availability statement

Data included in article/supplementary material/referenced in article.

### Declaration of interests statement

The authors declare no conflict of interest.

### Additional information

No additional information is available for this paper.
